# Patient experiences of occupational formulation: It's about getting to know me

**DOI:** 10.1111/1440-1630.70047

**Published:** 2025-09-01

**Authors:** Lorrae Mynard, Ellie Fossey, Louise Farnworth, Genevieve Pepin

**Affiliations:** ^1^ Department of Occupational Therapy Monash University Frankston Australia; ^2^ Department of Occupational Therapy Forensicare Melbourne Australia; ^3^ Centre for Forensic Behavioural Science Swinburne University Melbourne Australia; ^4^ Occupational Science and Therapy Deakin University Geelong Australia

**Keywords:** chart stimulated recall, co‐interviewing, collaboration, forensic mental health, lived experience, Model of Human Occupation, occupational formulation, peer interviewing, secure hospital

## Abstract

**Introduction:**

Occupational formulation is a synthesis of assessment information that describes a person's occupational participation and needs, to guide collaborative goal and therapy planning. This study aimed to explore patients' experiences of creating occupational formulations and reflecting on them in an Australian secure forensic hospital.

**Method:**

This qualitative descriptive study is part of a larger participatory action research project aiming to implement occupational formulation and evaluate its use within a forensic occupational therapy service. An occupational therapist researcher and lived experience worker co‐interviewed patients who had completed an occupational formulation. A semi‐structured interview guide and chart stimulated recall were used to support discussion with each participant about their occupational formulation. The audio‐recorded interviews were transcribed, and reflexive thematic analysis was conducted.

**Consumer and community involvement:**

Extensive consultation with members of the lived experience, occupational therapy and social work teams, and the hospital's consumer advisory group informed the design and implementation of this study.

**Findings:**

Ten forensic patients, including nine male and one female (aged 30s–60s) from four units, participated in interviews. Three themes were developed. The first, “a getting‐to‐know‐you piece,” describes how participants viewed the occupational formulation and the process of developing it. The second, “talking about losses and limitations” reflects the challenges within the forensic system that participants highlighted through creating and reading their occupational formulations, while the third, “valuing its potential” depicts how the occupational formulations were used and could potentially be used.

**Conclusion:**

Overall, participants viewed occupational formulation positively and valued being heard and understood. The approach benefited patients and the therapeutic relationship, supporting collaboration and potentially shifting power within the patient‐therapist relationship. The study also identified missed opportunities to support further reflection and collaborative progress review. Suggestions were made for strengthening the development, documentation, and review of occupational formulations and goals.

**PLAIN LANGUAGE SUMMARY:**

Occupational therapists try to learn about people's daily lives to understand what matters to each person and set therapy goals together. One way they do this is through a process called an *occupational formulation*. This is a written summary made with the person to show what they need and want in their life. This study looked at how patients felt about using occupational formulation in a secure forensic hospital. This is a new way of working, so we wanted to learn more about how it was used and what patients thought about it.

The study was planned with help from people with lived experience, occupational therapists, social workers, and the hospital's Consumer Advisory Group. An occupational therapist and a lived experience worker interviewed 10 patients about their occupational formulations. The interviews were recorded and written down. Researchers then looked at what the patients said to find common ideas.

Three themes were identified: getting to know the person, talking about losses and limitations, and how the formulation helped or could be helpful. Most patients said the process was helpful. It made it easier to work with their occupational therapist and talk about what was important to them. They also wanted more chances to check in on their goals.

Based on what we learned, we made suggestions to help therapists improve how they create and review occupational formulations with patients. More research could look at how this works in other places.

**Key Points for Occupational Therapy**

- Secure hospital patients valued contributing to an occupational formulation, with opportunities for sharing and learning.
- Using occupational formulation may support collaboration between occupational therapists and clients.
- Opportunities for further reflection and regularly reviewing goals together may optimise the use of occupational formulation.

## 1 INTRODUCTION

Patients' occupational opportunities are profoundly impacted by the physical and procedural restrictions of secure forensic hospital environments (Farnworth & Muñoz, 2009; Tomlin et al., 2018, 2020). These restrictions limit access to personal belongings, activities, relationships, and the outside world (Tomlin et al., 2018). Furthermore, forensic hospital admissions are involuntary in nature, and most patients have either been found not guilty by reason of mental impairment or are prisoners requiring mental health care. Accordingly, the term “patient,” rather than client or consumer, is used in this context. The dual responsibilities of upholding community safety and supporting patient recovery are challenging in secure settings as restrictive and risk management approaches are at odds with recovery‐oriented principles that emphasise empowerment and self‐determination (Tomlin & Jordan, 2022). Yet, the use of recovery‐oriented approaches is expected in both contemporary mental health care and occupational therapy practice (Commonwealth of Australia, 2013; Nugent et al., 2017; Occupational Therapy Australia, 2025; Tomlin & Jordan, 2022). While various recovery‐oriented approaches have been described, a systematic review by Leamy et al. (2011) identified five key recovery processes: connectedness, hope and optimism about the future, identity, meaning in life, and empowerment. These processes may support the work of occupational therapists in forensic hospitals as they seek to support patients' occupational participation despite the restrictions (Duncan, 2008).

Occupational therapists draw upon various approaches to bring an occupational focus to their work, such as using occupational history assessments or developing an occupational profile (American Occupational Therapy Association, 2020; Fisher, 2013). Occupational formulation (OF) is a type of case formulation approach using an occupational lens. Including it within the therapeutic reasoning steps for applying the Model of Human Occupation, Forsyth (2017) described OF as a process used by an occupational therapist to synthesise information gathered through assessment and document the client's occupational situation and needs. Brooks and Parkinson (2018) proposed a three‐part structure for constructing an OF comprising: (1) occupational influences, (2) occupational presentation, and (3) occupational focus. Subsequently, they published a comprehensive guide to implementing a Model of Human Occupation‐based approach to occupational formulation (Parkinson & Brooks, 2021). It guides occupational therapists to complete a comprehensive occupation‐focused assessment, and then to construct a written occupational formulation that addresses occupational identity (reflecting the person's viewpoint), occupational competence (reflecting the occupational therapist's viewpoint), and key occupational issues. Parkinson and Brook's approach emphasises the use of plain language and narrative writing within the OF to communicate the person's story in an engaging and compelling way. The development of an OF supports decision‐making and the negotiation of measurable occupational goals (based on the key occupational issues) to guide therapy. Shorter‐term goals are encouraged, with the intention of reviewing and negotiating subsequent goals collaboratively during therapy.

Parkinson and Brooks (2021) described occupational formulation as primarily developed to benefit the person (and/or their carer/s), with potential to enhance the therapeutic relationship, assist the therapist's reasoning and inform the team. There is little published about the application of their approach to occupational formulation, except the report of a satisfaction survey by Parkinson et al. (2011). They surveyed occupational therapists, the multidisciplinary team (MDT), patients, and carers following implementation of the occupational formulation approach in a mental health and forensic mental health occupational therapy service in northern England. Parkinson et al. reported that using this approach improved “person‐centred practice, understanding of occupational issues and needs, treatment planning and team working” (p. 151), consistent with key elements for promoting recovery described by Leamy et al. (2011). For example, participating in MOHO based assessments allowed patients to identify strengths and challenges faced, with OFs helping them to feel understood. As one patient shared: “The process enabled me to understand a ‘jumble’ of thoughts, and probing questions from [my occupational therapist] helped me talk and explain my feelings. It also assisted me in putting things into perspective, understanding priorities and setting targets” (p. 150). Others commented: “it helped me see where I'm going with my life”; and “I feel as if I was treated very much as an individual with my own personal needs addressed”; and that the occupational therapists “helped me to clarify what I need and how to go about it” and “helped me break tasks down into smaller, more manageable chunks” (p. 151). Hence, this survey indicated the potential value of using occupational formulation in practice and more recently, its use internationally has been reported (Brooks, 2023). Given the lack of research evidence to inform its use in occupational therapy practice, further exploration of occupational therapist and patient perspectives of occupational formulation is warranted.

In 2021, a forensic mental health occupational therapy team in Australia commenced a participatory action research project to strengthen their practice process by implementing and evaluating the MOHO‐structured approach to occupational formulation. Already familiar with using MOHO, occupational therapists received initial training in OF, a range of resources, and ongoing support over approximately 2 years to encourage use of OF as their routine approach for synthesising and documenting assessment information, using the identified needs and goals to guide therapy. They were provided with a structured template (based on Parkinson & Brooks, 2021) for this purpose, with headings of *Introduction, Occupational identity, Occupational competence, Key occupational needs, Summary statement*, and (initial) *Measurable occupational goals and planned action steps*. The current study commenced approximately 2 years after occupational formulation was first implemented in the service and addressed the research question: what are patients' experiences of creating occupational formulations and reflecting on them in a secure forensic hospital setting?

## 2 METHOD

### 2.1 Setting

Forensicare is the statewide provider of forensic mental health services in Victoria, Australia. The organisation delivers care within prisons, courts, and community services, and a secure hospital for people who experience serious mental illness and become involved in the criminal justice system. This study was conducted at Thomas Embling Hospital, which comprises acute, sub‐acute, rehabilitation, and transition units supporting inpatients at different stages in their recovery. Approximately 85%–90% of patients are male, with schizophrenia the most common diagnosis. At the time of study, seven units (128 beds) were open, and 14 occupational therapists provided services in these units.

### 2.2 Study design

The consolidated criteria for reporting qualitative research (COREQ; Tong et al., 2007) guided study reporting. As a part of the larger participatory action research project, members of the occupational therapy, social work and lived experience (LE) teams contributed to the development and refinement of the design of this study. It was also supported by the hospital's Consumer Advisory Group. Ethics approval was granted by Monash University (Project ID 31760).

A descriptive qualitative approach was selected to understand patients' experiences of OF. In‐depth interviewing, combining both “co‐interviewing” (two researchers interviewing jointly; Velardo & Elliott, 2021) and “peer interviewing” (lived‐experience peer as interviewer; Devotta et al., 2016) was chosen. This dual‐interviewing approach was selected in recognition of the power imbalance between practitioner researchers and patients in clinical settings (Devotta et al., 2016), as well as to enhance the quality of information sharing given participants may feel more comfortable speaking with an interviewer with a shared lived experience (Croft et al., 2016; Di Lorito et al., 2017; Lindquist‐Grantz et al., 2022; Velardo & Elliott, 2021). This approach also enables emotional support and learning opportunities for interviewers (Monforte & Úbeda‐Colomer, 2021; Velardo & Elliott, 2021) and may increase insights gained (Di Lorito et al., 2017; Velardo & Elliott, 2021).

### 2.3 Positionality statement

All researchers were female, with origins in Australia, Canada, New Zealand, and the United Kingdom. LM and BM were co‐interviewers. At the time of data gathering, LM was a doctoral candidate and experienced occupational therapist who had worked within the forensic service for 9 years, although not within the hospital itself for the previous 5 years. BM had been employed in the forensic service for 4 years as a lived experience worker, drawing on her personal experience of mental ill health to support and advocate for patients and advise on patient perspectives. LM had previous research interview experience; both LM and BM were experienced in conducting interviews in the study setting and had an awareness of the restrictions experienced by patients; neither was involved in the clinical care of study participants. The other authors are occupational therapy academics with qualitative research and mental health practice experience in varied settings, including extensive experience in forensic services (LF). The authors share interests in occupational formulation from long‐standing engagement in educating and supporting occupational therapists to use occupation‐focused approaches, such as MOHO, to guide their reasoning and practice. They also value seeking the lived experiences of people who receive occupational therapy services as central to guiding improvements in the quality and usefulness of these services.

### 2.4 Participants and sampling

A purposive sampling strategy (Liamputtong, 2020) was employed, with an anticipated sample size of 10–15. Eligibility criteria included that participants had previously developed an OF with an occupational therapist, had conversational English, were assessed as stable by clinical staff performing daily mental state examinations, and were able to provide informed consent.

Occupational therapists in the hospital identified patients who met eligibility criteria, offering them plain language information flyers. Twenty‐four patients from six units were identified by occupational therapists as meeting the inclusion criteria. The two interviewers (occupational therapist and lived experience co‐researchers) then arranged to visit the units at times that were suitable to meet eligible patients and provide them with the explanatory statement, answer questions, and invite participation. Ten patients provided written informed consent to participate in an audio‐recorded interview and access printed copies of the OF. Retail gift cards were offered as an acknowledgement of time and expertise.

Another ten of the eligible patients declined to participate, two expressed interest but became unwell, and two had not been invited to participate by the time data gathering concluded. In regard to sampling, Braun and Clarke (2021b) posit that data saturation is not theoretically consistent with reflexive thematic analysis, the approach used in this study. Arguing that sample size determinations include pragmatic considerations, such as available time and resources, they suggest considering information power instead, i.e. the more information relevant to the study question contained within the sample, the lower the required number of participants (Malterud et al., 2016). In this study, these pragmatic considerations and information power meant that when we noted after the ninth interview that similar ideas had been shared by multiple participants, we decided to conclude sampling following the tenth interview.

### 2.5 Data gathering

Shaped by input from the members of the occupational therapy, social work, and lived experience teams, a semi‐structured interview guide (see Table 1) was developed to balance flexible discussion with consistency of topics (Bowling, 2014). Plain language was used, and privacy was protected by seeking only broad demographic information. Questions covered the experience of creating and using an OF, were open‐ended, and included prompts to elicit further information (Liamputtong, 2020).

**TABLE 1:** Interview questions.

Questions	Prompts
Experience of creating an occupational formulation	
What do you recall about the process of developing this occupational formulation with your occupational therapist?	Participation in occupational therapy assessments/interview/discussion/writing the formulation/reviewing a draft/developing goals.
What do you like or not like about it?	Do you agree with what it says? Consider aspects from key sections: introduction/identity/competence/key needs/goals. Is there anything you learned about yourself through creating the occupational formulation?
Did you have a say in what was written?	In what way? What would you like to have happened?
Experience of using the occupational formulation	
Do you have a paper copy of your occupational formulation?	
Have you shared this occupational formulation with anyone else?	
How are you using the occupational formulation now?	Identifying recovery goals/Making plans for therapy/Supporting transition between units
On a scale of 0–10, how useful has the occupational formulation been? (0 meaning not at all useful, 10 being extremely useful).	Explain reason for selected number.
How could this occupational formulation be useful to you going forward?	
Overall reflections/feedback	
How would you describe to friend or family member what an occupational formulation is?	
What suggestions do you have for how the occupational therapy team could do better at developing occupational formulations with patients?	
Can you think of anything else about your experience of developing and using an occupational formulation that we have missed, or other thoughts that you would like to share?	

The chart stimulated recall technique was adopted to support participant recall. This technique involves reference to the medical record within a semi‐structured interview to provide a more detailed understanding than either the medical record or participant's description alone (Jennett & Affleck, 1998; Sinnott et al., 2017). Previously used for assessment of physicians' competency in patient interactions and care (Goulet et al., 2007), the technique has been adapted for use in research about care provision (Sinnott et al., 2017). In occupational therapy research, D'Cruz et al. (2020a) used a similar approach to support people with acquired brain injury during research interviews that involved reviewing participants' previously created written or digital narratives with them to facilitate recall. Hospital Health Information Management granted permission to print a copy of the OF from the electronic medical record to support participant recall during interviews.

Data were gathered over 8 weeks from January to March 2023. Interviews were conducted face to face in a private room on patients' units. Given COVID‐19 protocols, interviewers wore medical masks which did not seem to hamper interactions as patients were used to seeing staff wearing masks. To establish rapport, interviewers introduced themselves, the research and invited questions. Given the long‐term nature of forensic hospital admissions, some participants were known to either or both interviewers, which supported rapport, although neither were involved in their OF development or clinical care. To create a sense of shared experience with participants, at times BM spoke briefly about her own experiences of taking medication, experiencing memory difficulties and being in hospital. To support variations in literacy, participants were offered the choice of reading their formulation independently or having an interviewer read it out loud, and of interspersing reading by section with questions or reading the whole before responding to questions.

Together interviewers recorded detailed reflexive fieldnotes following each interview, including reflections as well as observations about the written OFs reviewed; participant engagement; and impressions of things left unsaid.

Recordings were transcribed verbatim using a secure automated transcription service, with amendment and anonymisation completed by LM and final revision by BM. Gender‐neutral pseudonyms were allocated and hospital units described by type. When offered, five participants chose to receive copies of their interview transcripts; none asked for adjustments.

### 2.6 Data analysis

Braun and Clarke's (2021a) six phases of reflexive thematic analysis guided the transcript analysis, with fieldnotes as complementary data, providing further context. Though presented sequentially, phases were iterative. Familiarisation with the dataset commenced with reflexive fieldnotes during interviewing. During transcription, both interviewers recorded reflections about individual interviews and the dataset. Following transcription, LM completed the familiarisation process individually due to BM's unexpected death. NVivo software supported use of an inductive coding approach: LM systematically added code labels, coding transcripts twice, in a different order each time (Braun & Clarke, 2021a). Regular review of fieldnotes and reflections supported understanding of the interview context and integration of BM's recorded perspectives. To support trustworthiness, care was taken to code what participants expressed, rather than sentiments indicating agreement with interviewers' comments or reflections. Initial themes were generated based on patterns observed within the codes, and relationships between themes considered. Themes were reviewed and further developed by re‐reading all the transcripts, associated fieldnotes and reflections to consider the fit between the data and the developing themes; the data within each theme was also reviewed to ensure consistency of concepts. Themes were refined, defined and named while writing up findings. Consistent with Braun and Clarke's (2021a) assertion that single researcher coding is acceptable within reflexive thematic analysis and need not be viewed as a limitation, LM led phases 2–6, with regular discussion of the coding and developing themes with other authors. BM's death meant that there was no LE input during the processes of re‐reading the transcripts, making additional reflections, coding or theme development. Nevertheless, the perspectives of her LE colleagues were sought when LM shared the written draft and met with three LE workers to discuss the findings. They viewed the findings as authentic from a LE perspective and provided suggestions for how to describe some elements, which were integrated into the final write‐up.

### 2.7 Supporting rigour

Consultations with the occupational therapy, social work, and lived experience teams informed the interview guide development. A co‐researcher with lived expertise was included to foster a more reflexive and collaborative research process (Veseth et al., 2017). Co‐researcher interviewing, use of reflexive fieldnotes, peer researcher discussions during coding and theme development, and feedback from lived experience team members aimed to ensure the findings represent the participants' perspectives.

## 3 FINDINGS

Ten forensic patients, nine males and one female, participated in interviews averaging 32 minutes (22–39 minutes). The proportion of male and female participants is reflective of the numbers of males and females within the forensic service. Demographic information and preferences regarding reading and keeping the OF are summarised in Table 2.

**TABLE 2:** Participant demographic information and interview preferences.

Characteristic	Value	Number
Gender	Male	9
	Female	1
Age	30s	1
	40s	6
	50s	1
	60s	2
Legal classification	Forensic patient	10
Length of stay	Years	4–35 (mean 12)
Unit	Postacute	1
	Continuing care	3
	Rehabilitation	3
	Transition	3
Time since OF	<1–3 months	8
	4–9 months	2
OF version	Initial	7
	Subsequent	3
Reading preference	Interviewer reading aloud	1
	Independent reading (in full)	3
	Independent reading (by section)	6
Keeping OF	Kept interview copy	9
	Already had a copy	1

Abbreviation: OF, occupational formulation.

Participants' recollections of developing and using an OF and initial goals were supported by the Chart Stimulated Recall approach. For example, Jude anticipated that reading the OF “might prompt my memory,” and Jac commented “that jogged my memory.” While recall was not prompted for two participants, reading the document enabled all participants to reflect on the content of the OF that they had created with an occupational therapist.

Three themes, shown in Table 3, describe how participants created and reflected on their occupational formulations and initial goals. The first theme “a getting‐to‐know‐you piece” describes how participants viewed the OF and the process of developing it. The second, “talking about losses and limitations” reflects the challenges within the forensic system that participants highlighted through creating and reading their OFs, and how these challenges affected occupational participation. The third theme, “valuing its potential” depicts how the OFs were used and could potentially be used.

**TABLE 3:** The three themes describing forensic patients' experiences of creating and reflecting on an occupational formulation.

A getting‐to‐know you piece	Talking about losses and limitations	Valuing its potential
A snapshot of who I am Influencing and trusting Reflecting and learning	I don't have that anymore It's quite limited Quest for a reasonable life	Sharing where I'm at Moving forward Looking back might help with staying on track

### 3.1 A getting‐to‐know‐you piece

This theme presents how participants described the OF document itself; their sense of influencing the process of its development while trusting the occupational therapist to authentically represent their perspective; and the opportunity for reflection and learning that the OF created.

#### 3.1.1 A snapshot of who I am

Participants described the OF as a summary bringing together information about different aspects of their lives, allowing others to get to know them, reflecting their progress, and as something to be proud of:

An occupational formulation is a bit of a in‐ insight into my progress … a snapshot of all different types of um … domains. (Kris)

A comprehensive summation of who I am as a person, um, my, including my goals and aspirations. (Jules)

It's a getting‐to‐know‐you piece with ah, some goal searching and challenges, but at the same time, some hope. Wear it as a badge of my hospital stay. (Jac)

#### 3.1.2 Influencing and trusting

Participants shared a largely positive sense of having influenced the development of the OF while trusting the occupational therapist to authentically document their perspectives in the written OF. First, participants viewed the process of creating the OF as directly shaped by information that they had provided. Jules remembered “sitting down doing the OCAIRS and MOHOST” (both MOHO‐based assessments), while other participants described “chats” or “interviews” about their life or work history, interests, activities, time use and future goals with an occupational therapist. Cam believed this process allowed the occupational therapist to get to know them, rather than relying on information from the medical record.

Some participants also described their active role in reviewing the draft OF with the occupational therapist and changing or endorsing the content. In Shannon's words, “we just went over the information and edited it,” while Jac noted making “a couple of changes” and a strong sense of agency in this process: “anything that does come up that I don't like, it can always be reviewed and deleted.” Finding reading difficult, Pat appreciated how the occupational therapist provided support by reading it out loud, “and l, I said, you know, that's fine. I agreed to, to the statements that had been made.”

Some OFs had also been developed or updated in preparation for care planning meetings or unit transition. For instance, Jac reflected on reviewing the previously developed OF with the occupational therapist after moving units as an experience of collaboration: “and then we decided to do another one.”

Participants also expressed satisfaction that the written OFs represented their voices and a sense of being understood. As Jules shared:

By reading it would seem that I've been able to have a fair influence. Um, either in quoted form or interpreted form of what I've said. So yeah, I feel that [occupational therapist] has captured quite well my point of view. … it captures a lot of the things that I say and think, but also insights into who I am as a person.

Similarly, Jude pointed out “I think it's been done word‐for‐word nearly!” The interviewers also observed the use of patients' words in quotation marks in many OFs, particularly in the occupational identity section.

Second, it seemed from their reflections that the participants viewed occupational therapists who completed occupational formulations with them as caretakers of their information, authentically documenting what had been shared and advocating for their interests. As Shannon articulated:

I like being able to put my faith and confidence in the competence of other people who have a role to play in my life going forward. And these, these words seem very competent. And like I can trust [occupational therapist], and I trust the words that have been written down about me, and for me, going, going forward. It's just such a good piece … I was able to read through it and think, “oh, good my future's in good hands here”.

Satisfaction with the overall accuracy of OFs was communicated through phrases like “it's pretty spot on.” Alex and Cam pointed out small factual errors related to admission dates and a goal respectively, but trusted that these could be corrected when the OF was updated, indicating they were not unduly concerned about these errors. Further, Cam linked the thoroughness of the document to the information gathered, believing the investment of time strengthened the process:

I think it's pretty much covered as well. Cause we went through it thoroughly with [occupational therapist] … it was more time consuming, but also it was very, I would say, thoroughly, thorough interview.

While most participants viewed the OF to be “comprehensive,” some noted sections where more contextual information could be added, such as about family supports, or forensic system challenges, or suggested that occupational therapists could seek additional perspectives and information from other staff.

#### 3.1.3 Reflecting and learning

Participants shared thoughts and feelings about the document and process of developing it, which included technical challenges and opportunities to learn, reflect, and plan. Technically, the length of paragraphs or the entire document was daunting for some, who were relieved by the opportunity to read and discuss it by section. The language used was inaccessible at times, causing uncertainty, with several participants requesting interpretation of words and acronyms. Some were confused by the presentation of key occupational needs in clinical language.

Developing the OF offered opportunities for self‐learning. Shannon made multiple references to the impact of seeing descriptions or circumstances “written down” or “in the text.” Indicating the impact of capturing information in writing, Shannon had the “eye‐opening” realisation that relaxation and enjoyment were difficult due to experiences of symptoms and guilt about the index offence; and reading the document informed Jules that others perceived them as competitive. Reflecting on the OF document could also bring feelings of affirmation. Kris thought the occupational identity section “sounded nice,” commenting it “made me feel good reading it,” and responded similarly to the occupational competence section, noting personal progress: “it's good. I like it. It says you know that I've gained more independence and understanding budgeting. Um, which is true.”

Reading about current challenges within the OF's occupational competence section could have been confronting, however none of the participants questioned the information documented. While Jac and Shannon found it difficult to read about symptoms and challenges, both agreed the descriptions were accurate. Franc expressed that the occupational competence section was “good at describing my negative symptoms,” which interviewers understood to refer to challenges broadly.

Jules articulated how the OF meaningfully brought together assessment information:

I think when you do tests like the OCAIRS and the MOHOST, it tends to be in a context of um bewilderment or ignorance, you don't really know how it's going to be used, you're not really sure of the process, you're not really sure of the outcome. I mean, when I look at a document like this, I think, “oh, so that's how it was used”. But prior to seeing the document you just think “oh, that's another bit of paper I filled out”, pretty meaningless. But seeing it in context, like this, you go “oh, this a useful undertaking.”

Stef too noted it was “good to um put words on paper” and recommended that patients should “participate in into it as much as you can.” Further, participating in the OF process provided direction to support further learning and planning, as Shannon explained:

You sit down with [the] OT [sic] and talk about how you're going, talk about your life, your past, present and future and talk about goals. And it's an enjoyable process and you can put down on, you can find some goals that are enjoyable to look forward to, to, to work towards, and you can reflect on some positives of your past. And also, there's some eye‐opening honesty with it. Yeah, yeah. You can get a sense of, get a reality check of how you're going. Like you can't, you won't get ahead of yourself and think you're going better than you are. It'll be an honest but *positive* reality check to go through.

… It gives you a sense of direction … And if you don't have a sense of direction, it'll help you find one. And helps with motivation. You see some goals, see what you've done previously, get some steely resolve in, how you're going about things and give some positive things to look forward to, and that's motivating as well.

### 3.2 Talking about losses and limitations

Participants reflected on losses and limitations that creating the OF had highlighted. The introduction and occupational identity sections of participants' OFs often contained reference to difficult and confronting life events such as mental ill‐health, homelessness, offending, and drug use. Referencing these elements of their lives, participants talked about their experiences of living within a secure setting. Losses due to offending and incarceration, and their current environmental restrictions limited opportunities for occupational participation and the type of goals that were possible to identify in the OF. Nevertheless, participants hoped for normal lives in the community, a quest that was not always evident in the OF.

#### 3.2.1 I don't have that anymore

In response to reading the OF, some participants openly referenced their offending and incarceration experiences and how these negatively affected their occupational lives. Some OFs directly articulated these losses; in others, these issues were implied in what was written. In both instances, participants were painfully aware of the resulting past, present, and future losses:

It can be quite difficult when you got a mental illness and, you know, you've committed an offence, it's not a good life. (Pat)

I had quite a good life outside of being incarcerated, prior to that I enjoyed playing [sport and working] … So, it makes me a little bit, a little bit sad that I don't have that anymore. (Shannon)

These challenges included not being free to live in the community, or to experience the ownership and life experiences of same‐aged peers, “like no super [retirement fund] … no holidays, no house, no car to talk about, and education” (Jude), as well as forced separation from family, inability to fully participate in parenting roles and lost friendships. Participants also anticipated re‐building lives and making new friends as challenging “cause people are quite judgmental” (Jude), leading to reliance on hospital staff and community support workers.

#### 3.2.2 It's quite limited

Being able to pursue meaningful occupational goals was something that participants noted was quite limited when they reflected on their OF. Participants described how the restrictions of the secure hospital shaped opportunities both within the hospital and the community, limited their power to make or enact decisions, and meant they were reliant on staff, hospital systems, and legal requirements to be able to pursue goals. They often mentioned being frustrated by the restrictions in units within the secure campus and being appreciative of or looking forward to lesser restrictions in the transition unit. Located outside the secure perimeter, this unit allowed access to resources such as the internet, mobile phones, or musical instruments without supervision, increasing autonomy, reducing dependence on others, and expanding opportunities for pursuing goals such as tertiary study. Cam noted that moving to this unit would allow greater scope for goals:

Being in [current unit] is quite limited, so I can expand all these things that I really wanna do in future goals, bus [sic] is just I need I need a little bit of freedom in a way.

Further, Jude highlighted how legal and hospital procedures for community access, such as day leave, could delay or disrupt options for community engagement. This put many voluntary and paid work roles “out of reach” (Jude), limiting opportunities to pursue activities aligned with interests, and reducing aspirations articulated in OFs to pragmatic options.

#### 3.2.3 Quest for a reasonable life

During discussing their OF, participants expressed a strong desire for a future life outside of the secure hospital, although this was not always reflected in their OF documents. For two participants, their desire was simply to leave the hospital, although they conveyed a sense that a better life was out of reach or of being unsure how to progress. Other participants expressed hopes in the interviews, not always reflected in the OF, that included obtaining stable, long‐term housing, gaining a driving licence and car, studying, working or volunteering, participating in fitness pursuits, and rebuilding family connections. Reviewing the OF goals recorded, Jac commented that “these are pretty tame goals, I'll need some meaty ones.” Jac wanted returning to driving to be included in the OF, feeling this might help to communicate its personal importance to the clinical team who were not presently supportive of it. Both Jac and Pat identified finding a home as a personal priority that was missing from their needs and goals. In contrast, Shannon described a “quest for a reasonable life” as their future hope but was unconcerned that some hopes were not included in the OF, feeling these would become more achievable when residing on a less secure unit.

### 3.3 Valuing its potential

Drawing on their experiences of the OF process, participants highlighted its potential value and the importance of ongoing review and engagement with the OF and goals to support its meaningful use. Specifically, participants viewed the OF as a useful “tool” for communicating information about them as individuals to others and for supporting them in goal striving or moving forward, whilst also pointing to the need for more opportunity for reflecting to affirm a sense of progress.

#### 3.3.1 Sharing where I'm at

Participants understood that the completed OF document was stored in the medical record and had potential to communicate information to others. Jac thought staff could look at it to “get a gauge of what … kind of person I am, as a consumer.” Pat appreciated the benefit of information being documented:

instead of it just bein' in my thoughts it's on paper and now the doctors and my team can see, you know where I'm at, 'cause … they can't read my mind.

The document could be useful in care planning meetings “where you sit and talk about your goals … with the staff” (Shannon). Other identified uses included supporting National Disability Insurance Scheme [community support] funding applications or sharing information with family members.

#### 3.3.2 Moving forward

All participants described moving forward towards initial goals within their OFs by working together with an occupational therapist or other staff on individual pursuits, such as structuring a routine, preparing a resume, building family connections, or participating in groups focused on skill development, interests, and social interaction. Some participants also described pursuing goals independently (e.g., exercising and enrolling in study), and several noted limited staff capacity for following up needs and goals, making observations about understaffing or workloads of nurses, social workers, and occupational therapists.

Some participants noted that their actions taken towards moving forward on some goals had differed from those recorded within the OF because of changed circumstances, new information, or the documented steps not reflecting their preferences. For example, although indicating agreement with their direction, Kris did not recall contributing to goal development and disliked the action steps described for some goals, suggesting they reflected staff preferences rather than Kris' own. Further, Kris indicated they were keen to work towards their identified goals by pursuing different activities.

#### 3.3.3 Looking back might help with staying on track

During their interviews, several participants noted changed circumstances since the OF was documented, identifying elements they would update if rewriting the OF, although the key occupational needs remained relevant. Despite this, only one participant reported having re‐read their OF or goals with an occupational therapist. Having done this after moving to a new unit, Jac stated a desire to revisit goals “after three, six months … [and] … maybe make some more goals.”

Pat and Kris highlighted the value of reflecting on their OF during the research interview, and suggested that regularly reviewing their OFs could assist them to recognise their progress:

“I could just see where I was at the time of this and where I am now, it's, I like seein' the change, the full positive.” (Pat)

I would never really gone back and looked at it. And so, I don't know maybe if ah staff would prompt you to look at it once every six months, or, or as frequently as they think, you know. Just go, “oh yeah, I am on the right track”. (Kris)

## 4 DISCUSSION

This is the first known study to investigate the experience of OF from patients' perspectives. Three themes describe how participants residing in a forensic hospital created and used OFs with an occupational therapist. Participants described occupational formulation as “a getting‐to‐know‐you piece,” sharing their experiences of influencing its content and development. When “talking about losses and limitations,” participants reflected that occupational possibilities and goals were shaped by the forensic setting. In the theme “valuing its potential,” participants shared about how their OFs were being used and could potentially be used.

Overall participants viewed the process of creating and reflecting on an occupational formulation positively, although opportunities to strengthen its use in practice were also apparent. The process of collaborating to develop the written OF, and its use in supporting reflection, learning, and planning will be discussed, before offering suggestions for practice.

### 4.1 Occupational formulation as an opportunity for collaboration

Consistent with the recovery process of empowerment (Leamy et al., 2011), collaborating with occupational therapists to develop written occupational formulations allowed patients to communicate their perspectives in a secure hospital setting where they had little choice and control over much of their lives. Reflecting on the written narratives, participants appreciated that their voice was included, as indicated by their words being used and their stories shared. This aligns with Parkinson and Brooks's (2021) assertion that occupational formulation exists to give a voice to the person. As forensic patients have not traditionally contributed to the development or review of health record documents, collaborating on these processes unsettles the therapist‐patient power imbalance. With limited opportunities to exercise power themselves, collaboratively developing an OF with an occupational therapist considered trustworthy and competent was seen as beneficial to their stories being shared and their interests advocated (without needing to repeat themselves) within the medical record, team meetings, or with family members.

The written OFs did not always reflect participants' aspirations for their future lives. This could be because they spoke little of these topics when developing their OFs. Potentially, the occupational therapists may not have asked about bigger hopes or thought it necessary to include longer‐term needs within the OF. Perhaps fearing that inclusion of needs such as “getting a driving licence” might communicate false hope, occupational therapists may have focused on documenting needs and goals that they deemed more likely to be endorsed by the MDT or more achievable within the scope of service delivery. This suggests potential tensions between collaboration in practice and systemic aspects of the setting, and that ongoing and intentional use of a collaborative approach for occupational formulation is required if patient perspectives, including their big hopes or dreams, are to be authentically reflected within the OF narrative. Similarly, Nugent et al.'s (2017) study of occupational therapists' experiences of recovery oriented practice identified the need for active commitment and ongoing reflection to develop and sustain a recovery orientation in mental health practice, as well as advocacy to bring about systemic change.

Opportunities for strengthening collaboration to enhance patients' contributions in the development of OFs were also identified. Overall, the language of the occupational identity and occupational competence sections of OFs was described as accessible by participants, but the use of clinical language at times (often related to key occupational needs) caused some confusion. This suggests that while overall the OFs were written in plain language with the understanding that they would be read by the person, as recommended by Parkinson and Brooks (2021), further attention may be needed to describe occupational needs using the person's words, as these form the basis for negotiating goals and actions in therapy.

Since participants offered more critique of goals and action steps than of other sections of the OF, they may also have contributed less to their development. Inviting and supporting participation in the negotiation of goals is encouraged in Parkinson and Brooks' (2021) occupational formulation approach, and can enhance engagement in ongoing therapy (Conneeley, 2004). Supporting goal striving is also consistent with adopting a recovery‐oriented approach in mental health practice (Slade, 2013).

Furthermore, participants' identification of factual errors, contextual information that could be added, and concerns relating to needs and goals led us to wonder how invested or equipped they had felt when invited to review the draft OF. Jac's view that they were able to delete information not reflecting their perspective shows a strong sense of agency but also highlights the potential risk of OFs being incomplete, inaccurate, or less useful if occupational therapists remove *any* content a person does not like, rather than ensuring inclusion of factual information about the person's occupational competence. Using a structured draft review process could support collaboration on the document and provide an opportunity to explore differing perspectives, as well as offering people time and support to understand wording, clarify meaning, and share suggestions. Similar to the findings of D'Cruz et al. (2019, 2020b), patients in this study valued the honouring of their stories through the creation of an OF that stayed true to what had been shared with the occupational therapist, and their involvement in its review and refinement supported learning.

### 4.2 Supporting reflection, learning, and planning

The process of developing OFs allowed patients and occupational therapists to reflect, learn, and plan together. Reflection on occupational identity and occupational competence often brought a sense of feeling affirmed and could lead to learning. Consideration of their progress and potential supported a sense of direction and motivation, with the written OF serving as a map or tool for moving forward. Seeing the written OF and initial goals brought meaning to the process of doing assessments.

These findings are consistent with patient feedback from Parkinson et al.'s (2011) satisfaction survey, in which patients valued being seen as individuals through the use of OF, as well as having opportunities to reflect, learn about strengths and challenges, and consider future plans. Similar findings were identified in an exploration of the perspectives of community mental health service users and occupational therapists about the use of the Occupational Performance History Interview (OPHI‐II; Ennals & Fossey, 2007). Service users in that study also appreciated being able to talk about things that mattered to them, beyond medical care, illness, and risk, which included their joys, challenges, and future hopes. Further, D'Cruz et al. (2019, 2020a, 2020b) reported that the use of narrative approaches contributes to building a strength‐based identity, through communicating with others to process feelings, losses, and challenging situations, and through being validated by having someone listen. Aligned with their conclusions, the current study indicates that patients in a forensic hospital valued conversations about their occupational lives that allowed for reflection on change and progress, affirmation, and consideration of future directions.

Gathering information for the OF can help to contextualise a person's occupational participation (Parkinson & Brooks, 2021). For participants in this study, OFs reflected the difficulties faced because of mental illness and involvement in the forensic system. When reading their OFs during interviews, participants acknowledged challenging information about their history or occupational competence, with some reflecting on how this led to learning. While it may feel confronting, sensitively discussing difficult elements of people's occupational stories may support their learning. Similarly, giving feedback on occupational performance is a strategy that can support a person's learning and ultimately their occupational participation and engagement (Pepin, 2024). Fossey et al. (2002) also reported that sharing feedback with consumers can foster hope and facilitate learning, and offered therapists recommendations for providing sensitive and strengths‐focused feedback. Similarly, the use of OF may provide a helpful framework for occupational therapists to raise difficult topics and provide feedback in a manner that supports recovery. That most participants in this study had not been invited to review their OFs and goals represented missed opportunities for reflection on progress, learning, and celebration of achievement, but also for revising goals and actions consistent with the OF process described by Parkinson and Brooks (2021), which anticipates that sequential goals will be developed over time towards the identified occupational needs. The long‐term nature of forensic hospital admissions may have reduced occupational therapists' sense of urgency for regular goal review; yet regular review is essential to support the focus of ongoing therapy and to identify when key occupational needs have been addressed or are no longer relevant, triggering an update of the OF.

### 4.3 Suggestions for strengthening the use of occupational formulation in practice

Participants' experiences of occupational formulation were largely positive. Their trust of occupational therapists to represent their information through an OF is both encouraging and sobering, indicating the value of and responsibilities associated with using the approach. Training and supervision may support occupational therapists in developing and refining skills for collaborative development of occupational formulations. Informed by the findings, Table 4 offers suggestions for how occupational therapists could strengthen their use of OF and collaborative practices.

**TABLE 4:** Suggestions for strengthening use of occupational formulation in practice

Suggestions for occupational therapists to strengthen use of OF
Address potential collaboration barriers, e.g., symptoms, medication side‐effects, trauma histories and personal or systemic challenges Explain the OF process clearly, adapting steps to individual needs (e.g., session length and reading support) Include the person's hopes or dreams in the occupational identity section, even if needs/goals are limited by service constraints Negotiate goals together, focusing on short‐term goals to allow engagement in change Write the OF in plain language, including the person's words Structure the draft review process to allow two‐way feedback Offer a copy of the OF where possible Conduct regular collaborative goal reviews

### 4.4 Strengths and limitations

A commitment to stakeholder participation supported the development of a robust study. With the time between OF development and interview ranging from less than 1 to 9 months, longer gaps may have limited participants' ability to reflect on the OF process. The use of chart stimulated recall was intended to support recall in this context. Conversely, those with a shorter time between development and interview had less experience of using the OF on which to reflect. Co‐interviewing by an occupational therapist researcher and LE worker was intended to reduce unequal power between patient‐participants and staff‐researchers. It supported participants' comfort when sharing their experiences, enabling the collection of rich data representing the perspectives of participants at different points in their recovery journeys. Participants' willingness to identify errors and/or share negative perspectives suggests that interviewers' attempts to frame questions neutrally and demonstrate openness to hear genuine experiences were effective. Coding focussed on what participants expressed rather than agreement with interviewers' comments. Additionally, BM's lived experience‐informed insights (in fieldnotes and reflections) enhanced understanding of the data, and seeking input from other LE colleagues ensured inclusion of the LE perspective despite her death. Although member checking with participants to ensure the authenticity of findings was limited, a plain‐language summary of findings was developed with input from a LE colleague, who accompanied LM in visiting units to share it with participants. Trustworthiness was supported by the use of a detailed audit trail throughout the study, reflexive fieldnotes to support analysis, and thorough presentation of the study using the COREQ (Tong et al., 2007). While participants were forensic patients within one hospital, elements of the findings may be transferable to other settings.

## 5 CONCLUSION

This study explored patients' experiences of creating an occupational formulation with an occupational therapist in a forensic hospital setting. Overall, participants viewed occupational formulation positively. Consistent with previously reported benefits of using OF, the findings reflect important gains for the person and the therapeutic relationship: creating opportunities for the person to be heard and understood, shifting power towards more collaborative working, and supporting learning. Such elements offer a strong foundation for meaningful, occupation‐focused, and recovery‐oriented occupational therapy practice. However, opportunities were missed to build on the strengths reflected in the development of the occupational formulation through supporting more collaborative reflection and regular shared review of goal progress. Suggestions were made for optimising the use of the OF approach. Future research could explore experiences of OF for other populations, occupational therapists, and in multidisciplinary teams. Longitudinal studies are also needed to explore the impact of using occupational formulation to guide occupational therapy services and outcomes.

## AUTHOR CONTRIBUTIONS

Lorrae Mynard led study design, data gathering, analysis, interpretation, and manuscript preparation, with regular review and input from Ellie Fossey, Louise Farnworth, and Genevieve Pepin.

## CONFLICT OF INTEREST STATEMENT

Lorrae Mynard is the founder of OccupationalFormulation.com, which provides occupational formulation training.

## Data Availability

The data that support the findings of this study are available on request from the corresponding author. The data are not publicly available due to privacy or ethical restrictions.
